# Public attention related to epidural labor analgesia in mainland China: Evidence from internet search data

**DOI:** 10.1097/MD.0000000000045978

**Published:** 2025-11-14

**Authors:** Xinzhu Zhu, Xu Wang, Xuetong Zhang, Peiwen Jia, Guannan He, Fengshou Chen

**Affiliations:** a Department of Anesthesiology, The First Hospital of China Medical University, Shenyang, Liaoning Province, China; b Department of Anesthesiology, Benxi Central Hospital of China Medical University, Benxi, Liaoning Province, China; c Department of Laboratory Medicine, The First Hospital of China Medical University, Shenyang, China.

**Keywords:** Baidu index, epidural labor analgesia, search engine

## Abstract

The Baidu index (BDI) is a publicly available data platform that reflects search volume and user attention trends based on massive behavioral data from its users. Utilizing this tool, this study aimed to investigate the public search trends and level of understanding regarding “epidural labor analgesia (ELA)” in mainland China. Data were collected from January 1, 2016 to October 16, 2024, to analyze search volumes and related search terms. The influence of 2 pivotal events was also assessed: the issuance of the government’s “Notice on Labor Analgesia Pilot Work” promoting ELA, and a widely publicized maternal suicide incident linked to unrelieved labor pain. Search volume for ELA showed a fluctuating upward trend from 2016 to 2018, followed by a steady decline from 2019 that persisted until 2024. The release of the “Notice” was associated with a statistically significant increase in the BDI (*P* < .01). Conversely, following the maternal suicide incident, the average BDI for ELA demonstrated a significant decreasing trend (*P* < .01). Analysis of the top related search terms revealed that public attention was predominantly focused on general childbirth themes, such as alternative delivery methods (“episiotomy”) and the intensity of “pain experienced during childbirth.” In contrast, searches for ELA-specific themes, including “the effects of ELA,” “the cost of ELA,” and “why not promote ELA,” were relatively scarce. This pattern indicates a limited and nonspecific public understanding of ELA. In conclusion, the BDI proves to be a valuable infodemiology tool for monitoring public interest in health topics. The identified gap between general childbirth queries and targeted ELA searches highlights a significant knowledge gap. These findings underscore the urgent need for targeted health education campaigns and supportive policy initiatives to improve public awareness and acceptance of epidural analgesia, thereby facilitating more informed decision-making during childbirth.

## 1. Introduction

Labor pain is a natural part of normal vaginal birth and can be caused by vaginal examinations, uterine contractions, and vaginal lacerations.^[1,2]^ Most women experienced extremely severe pain, during childbirth. Labor pain ranks first in pain scoring, though pain is the fifth vital sign.^[3]^ Epidural labor analgesia (ELA), an important development in modern fertility care, is currently the most reliable and widely applicable childbirth analgesia recognized by the international anesthesia community.^[4]^ In 1847, ether was first used for difficult vaginal delivery, marking an important moment in the history of obstetric anesthesia.^[5,6]^ Many effective pharmacological and non-pharmacological methods are available for managing labor pain, such as morphine and diamorphine, epidural analgesia, nerve blocks, transcutaneous electrical nerve stimulation, relaxation, and massage.^[7,8]^ Due to fear of pain, labor analgesia may prevent women from choosing cesarean delivery (CD), which is more conducive to maternal and neonatal safety. Epidural was found to offer better pain relief, a reduced risk of acidosis in the newborn, and a reduced risk of naloxone administration in the newborn.^[9]^ ELA also represents humanized medical service and advanced fertility care in modern society. In addition, ELA can reduce postpartum depressive symptoms and improve short-term pelvic floor dysfunction.^[3,10]^ In Norway, epidural analgesia was used in 34% of all childbirths in 2018. Additionally, in Norway and other western countries, the use of epidural anesthesia during childbirth is still increasing.^[2]^ In the United Kingdom, the United States, Germany, and other developed countries, the rate of ELA is as high as 85% to 90%.^[11–14]^ Given that labor pain management is a central issue for maternal health worldwide, improving access to and understanding of ELA is of global public health relevance. Monitoring public attention to this topic can provide policymakers with valuable signals about unmet needs and inform the allocation of healthcare resources. In China, the rate of ELA differs by region. For example, the ELA rate in Eastern China is 30.77%, but is as low as 1.02% and 7.56% in Northwest China and Southwest China, respectively.^[15]^ The rate of ELA in Northeast China, where our hospital is located, is 11.65%. In large maternity hospitals and maternal and child health centers in economically developed areas, the ELA rate is much higher than that in second- and third-tier cities, where some hospitals have not yet carried out ELA. The current situation of ELA in China is not optimistic. Given that labor pain management is a central issue for maternal health worldwide, improving access to and understanding of ELA has major global public health relevance. Monitoring public attention to this topic can provide policymakers with valuable signals about unmet needs and help guide healthcare investment and resource allocation, both in China and internationally.

The development and public awareness of ELA in China have been significantly influenced by 2 pivotal events. On August 31, 2017, a widely publicized maternal suicide occurred in Yulin, Shaanxi Province, after a pregnant woman’s request for a cesarean section was denied amid severe labor pain. The incident triggered intense national debate on pain management and maternal rights, likely heightening online searches for ELA. The Chinese government is committed to increase the practice of ELA. The *Notice on carrying out Labor Analgesia Pilot Work* was issued by the National Health Commission on November 20, 2018,^[15,16]^ which launched pilot programs across China to promote standardized use of labor analgesia and improve maternal care. As an authoritative national policy, the Notice likely drew significant public attention and contributed to increased online searches for ELA.

Advanced internet technology is accessible to the public for information collection, release, dissemination, and acquisition, enabling individuals to be quickly informed and to educate themselves. Meanwhile, the search data of internet users are also recorded. Among 989 million internet users in China, according to the *47th China Statistical Report on Internet Development*, 81.3% use search engines to seek health information.^[17]^ Approximately 90.9% of internet users chose Baidu as their first searching choice. Currently, Baidu is the leading search engine in mainland China.^[18]^ The Baidu index (BDI) is a data-sharing platform based on massive amount of data on the searching behavior of its internet users, providing search indexes that are updated daily.^[19,20]^ The BDI reflects the active searching needs of internet users and the searching contents. It can detect the attention of internet users to specific keywords, which may help to discover new research topics and ideas in different fields. Furthermore, the BDI can provide real-time data on several diseases including kidney stones,^[18]^ premature ejaculation,^[21]^ lower urinary tract disorders,^[22]^ influenza,^[23]^ erythromelalgia,^[24]^ dengue fever,^[25]^ norovirus,^[26]^ and AIDS.^[27]^ However, the BDI has not been extensively applied in the health care field. This leaves a large amount of data that still need to be extracted and analyzed, including the searching behaviors and interests of Chinese users regarding ELA.^[18]^

In the present study, we aimed to obtain the internet users’ search data related to the term “ELA” through the BDI, and to analyze the characteristics of online public attentions using attention trends, temporal and spatial distributions, and population attributes. Furthermore, we sought to explore the public concerns regarding ELA by identifying and analyzing related search terms. We also evaluated the impact of both the maternal suicide incident and the national policy notice on public interest in ELA. Furthermore, our analysis provided information that is helpful to the promotion and future implementation of ELA.

## 2. Materials and methods

### 2.1. BDI data and netizen information

Baidu accounts for more than 80% of the internet market share, serving as the preferred search engine for Chinese internet users.^[28]^ Its subsidiary, the Baidu Search Index (BDI, http://index.baidu.com), a public data platform similar to Google Trends, parallels in aggregating keyword search data, and offering a window into public interest freely. Through the BDI, people can find search trends for selected terms, gain insights into changes in netizens’ needs, monitor media trends, and identify user characteristics.^[29]^ The BDI can be used as a data source to reflect the search behaviors of Baidu users. To explore public interests in ELA, the weighted sum of search frequency for relevant keywords was calculated and analyzed in a specific area for a preset period using the BDI. The BDI is presented as an index value rather than raw search counts. It is generated by Baidu through a proprietary algorithm that weights search frequency, such that higher values correspond to greater relative search interest, but without a defined physical unit of measurement. The demographic data, which displays the gender and age distribution of users who searched for the keyword, is generated as follows: based on Baidu user search data, a computational clustering analysis is performed on the population attributes of the keyword to produce distributions and rankings for age and gender. Demographic attributes (e.g., age and gender) and keyword associations are estimated by the platform through these algorithms, and this study reports only the outputs provided by BDI. The more searches were conducted, the higher the search index was, thus reflecting increased public attentions.^[20]^ In addition, mean BDI was calculated to obtain the average attentions of internet users (1/100 million) in certain cities and provinces of China.^[30]^

This study was considered exempt by the institutional ethical committee of the First Hospital of China Medical University owing to the use of aggregate, fully anonymous, de-identified, publicly available search engine usage data. Information on the keyword “ELA” was collected from the BDI platform for the period between January 1, 2016 and October 16, 2024. The search for BDI data related to labor pain relief was completed on October 17, 2024. The same timeframe was consistently applied across all analyses, including comparisons between demographic groups and evaluations of the 2 major events (the 2017 maternal suicide incident and the 2018 Notice on carrying out Labor Analgesia Pilot Work). This alignment ensures that the results are directly comparable across sections.

**Figure 1.**
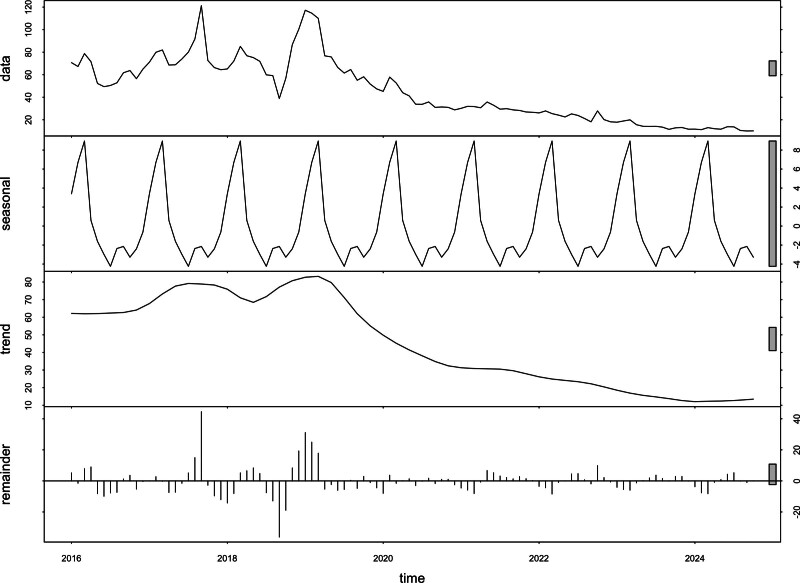
Time-series analyses were conducted on the monthly average value of BDI (Baidu index) for “ELA” (effects of epidural labor analgesia) from 2016 to 2024. The analysis included the breakdown of the data into trend, seasonality, and random components. BDI = Baidu index, ELA = epidural labor analgesia.

**Figure 2.**
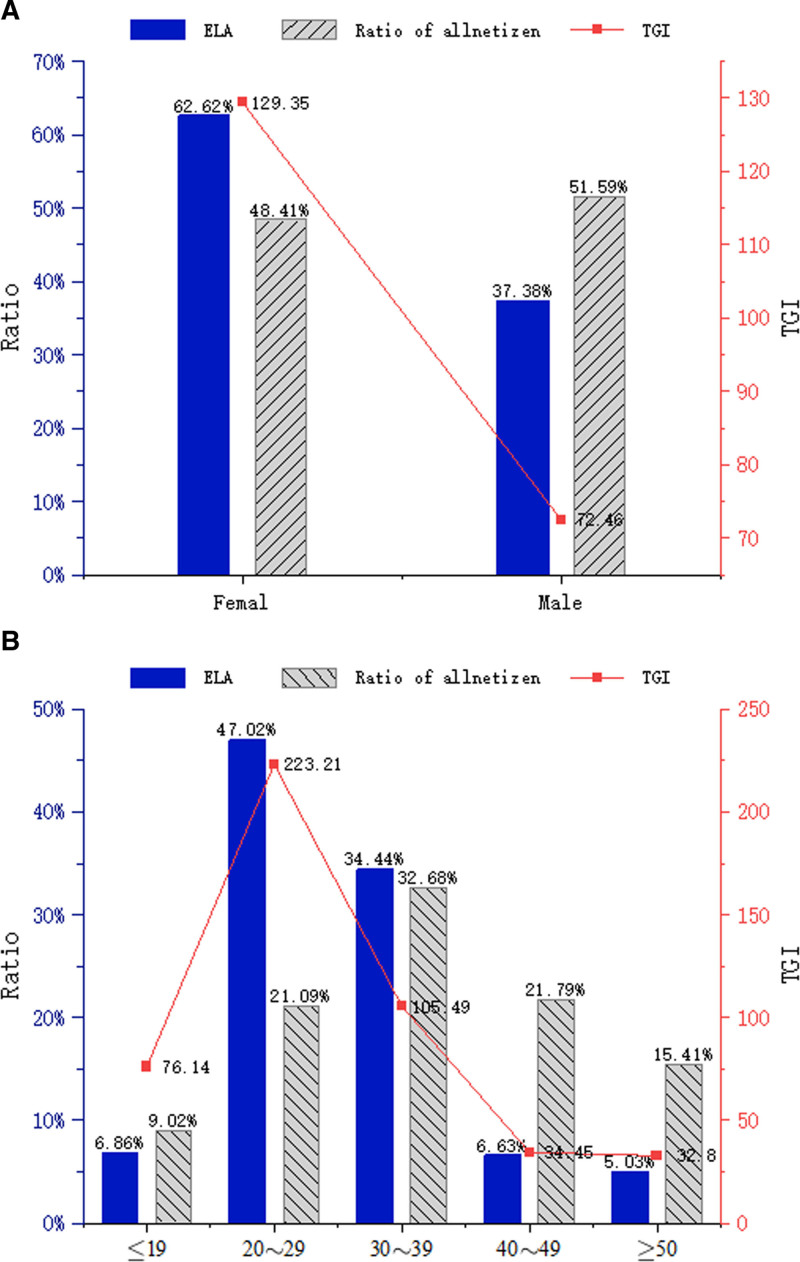
The demographic profiles of ELA searches by gender (A) and age (B). ELA = epidural labor analgesia.

The following data were extracted: daily search index values for the keyword “ELA” at the city, provincial, and national levels; user profile data (age and gender) provided by BDI (Fig. 1); and demand graph data showing the top 20 and top 10 most frequently co-searched terms with ELA (Fig. 2). The search volumes and demand graphs for “ELA” from January 1, 2016 to October 16, 2024 were recorded at the city, provincial, and national levels. Daily search counts based on gender and age were also extracted. Specifically, we compared search interest of ELA across gender and age groups by target group index (TGI).^[31]^ The higher the TGI score, the greater the interest of a target group in the search object.^[32]^

**Figure 3.**
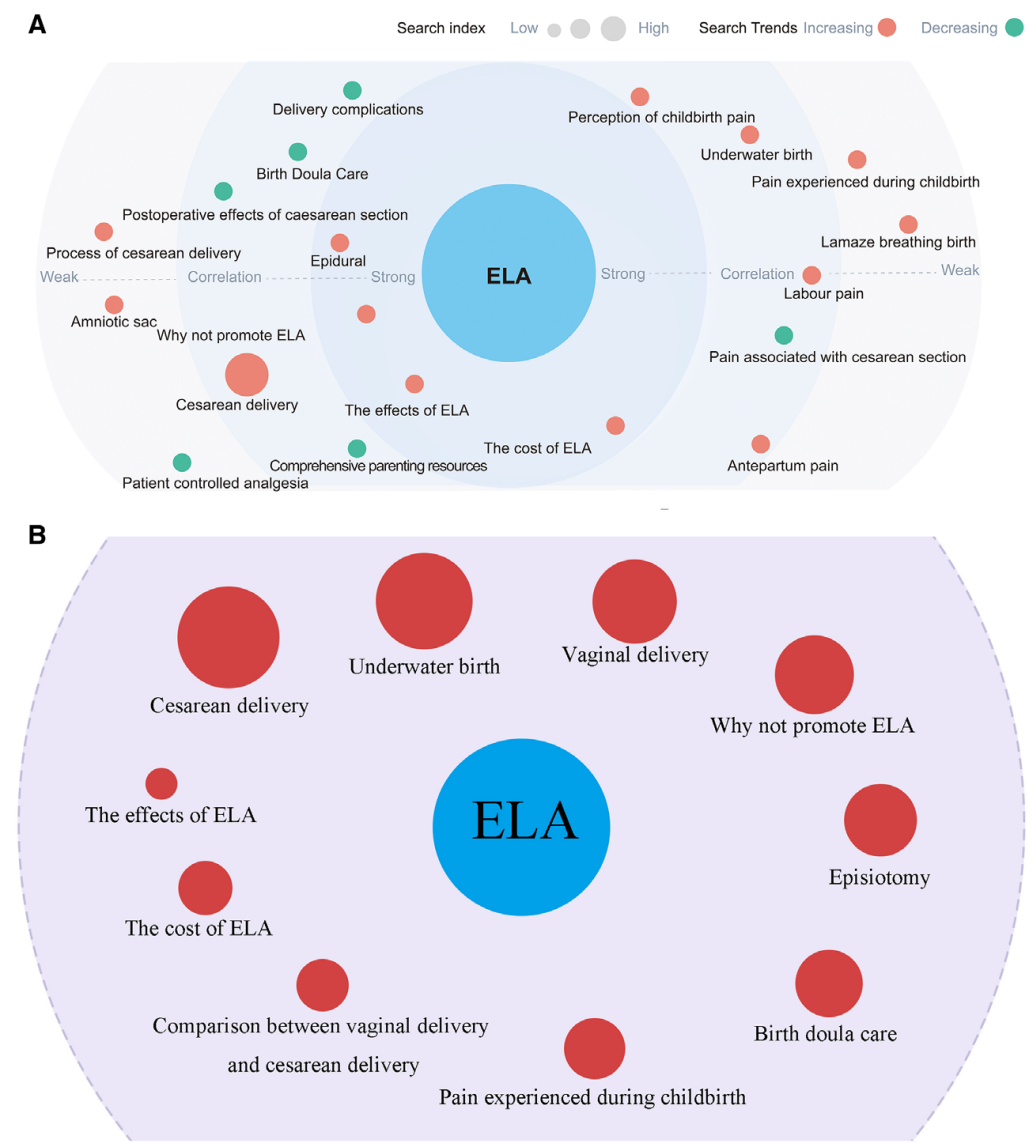
(A). Demand graph of ELA. (B). The top 10 high-frequency terms search terms associated with ELA. The larger the red color circle, the higher the frequency of search terms related to ELA. ELA = epidural labor analgesia.

### 2.2. Analysis of search terms correlated with ELA

To investigate public interest in ELA, the top 10 related search terms were obtained weekly throughout 2022 from the BDI platform’s demand graph. The frequency of each related search term was calculated. The top 10 search terms correlated with ELA were selected to assess different internet-seeking behaviors of internet users.^[33]^ We further analyzed the relationships between the top 10 related search terms ELA and ELA to better understand the search behavior of users.

### 2.3. Influences of the notice and maternal suicide due to childbirth pain on ELA-related public concerns

Based on the execution date of the Notice by the National Health Commission (November 20, 2018), the following 2 6-month periods were defined: period 1, from May 20, 2018 to November 19, 2018; and period 2, from December 21, 2018 to June 19, 2019. The time frame from November 20, 2018 to December 20, 2018 was excluded from this study to avoid the impact of extreme data, since the Notice was carried out on November 20, 2018 and aroused significant attentions in China over the following month.^[34]^ Moreover, we also evaluated the influence of maternal suicide due to childbirth pain. Based on the date of the incident (August 31, 2017), the following two 6-month periods were defined: period 3, from March 1, 2017 to August 30, 2017; and period 4, from October 1, 2017 to March 31, 2018. The influence of each factor was evaluated by comparing the mean BDI during 6 months before and after the 2 incidents, respectively.

### 2.4. Statistical analyses

This study used SPSS 19.0 for statistical analysis. The normality of the BDI data was assessed using the Kolmogorov–Smirnov (K–S) test. As the data for most periods were not normally distributed (as detailed in the Results section), the Mann–Whitney *U* test was employed to compare search volumes between different periods. Descriptive statistics are presented as median values with interquartile ranges (IQRs) rather than mean and standard deviation, as the median provides a more robust measure of central tendency for skewed data. A *P*-value of <.05 was considered statistically significant. Results were presented as median (IQR).

## 3. Results

### 3.1. Search trends for the term “ELA”

Using the period from January 1, 2016 to October 16, 2024 as the statistical time frame, we extracted the daily value of BDI for “ELA” from the BDI search platform (Fig. S1, Supplemental Digital Content, https://links.lww.com/MD/Q685). As noted in Section 2, the BDI is expressed as an index value rather than raw search counts. For demographic attributes such as age and gender, the platform applies proprietary clustering algorithms, and this study reports those outputs directly without any intervention or influence on the research data. We obtained the monthly average value of BDI for “ELA” for time-series analyses.^[35]^ The analyses revealed that from 2016 to 2018, the search volume exhibited a fluctuating upward trend over time, followed by a decline in the first half of 2018 and subsequent growth. However, after 2019, the search volume displayed a downward trend over time. By 2024, the overall interest in ELA (as reflected by BDI) appears to have settled at a relatively subdued level compared to the earlier peak years (Fig. 3).

### 3.2. Spatial distribution characteristics of ELA

A statistical ranking of the provinces in China by attention to ELA from January 1, 2016 to October 16, 2024, revealed pronounced geographic disparities across China. At the provincial level, Guangdong exhibited the highest search index, followed by Shandong, Jiangsu, Zhejiang, and Henan, then by Sichuan, Beijing, Hebei, Anhui, and Hubei, ranking 6th to 10th. For the 7 geographic regions of China, Eastern China showed the highest degree of attention, followed by Central China, Southern China, Northern China, Southwest China, and Northwest China. Northeast China had the lowest public attentions to ELA. As for the major cities of China, Beijing showed the highest public attention to ELA, followed by Shanghai, Guangzhou, Chengdu, Hangzhou, Shenzhen, Wuhan, Zhengzhou, Chongqing, and Suzhou. A total of 80% of the regions with a high search index were located in Eastern China, which had high urbanization and rapid economic development. These findings underscore that public demand for ELA-related information is spatially uneven, with significant overrepresentation in economically developed and highly urbanized regions. Consequently, these results highlight the need for targeted health communication strategies and equitable dissemination of maternal health knowledge, particularly in less-developed regions such as Northwest and Northeast China.

### 3.3. Demographic characteristics

Demographic data was obtained using the Crowd Portrait module of BDI. Analysis by gender revealed that females constituted the majority (62.62%) of users searching for “ELA,” with a TGI of 129.35, indicating a strong targeting tendency and pronounced gender-specific interest, compared to 37.38% males (TGI: 72.46; Fig. 2A). This distribution diverges significantly from the overall male-to-female netizen ratio in China (51.59:48.41), highlighting that ELA is a topic of particular relevance to women. Additionally, 47.02% of the internet users in our study were 20 to 29 years (TGI: 223.21), while 34.44% were 30 to 39 years (TGI: 105.49). Of the total sample, the number of individuals aged 19 years or below ranked third (6.86%; TGI: 76.74), those aged 40 to 49 ranked fourth (6.63%; TGI: 34.45), and those aged 50 and above ranked fifth (5.05%; TGI: 32.8; Fig. 2B). Exhibiting a remarkably high TGI of 223.21, the 20 to 29 age group paid the most attention to ELA, establishing it as the core demographic driving online attention. These findings clearly identify young adults (20–29 years) as the primary population seeking information on ELA, which aligns with the typical childbearing age range and underscores their heightened awareness and proactive health information-seeking behavior regarding childbirth options.

### 3.4. Characteristics of relevant search terms

The top 20 most frequently searched terms related to ELA (excluding “ELA” itself) on the Demand Graph of the BDI platform (Fig. 3A) were collected weekly throughout 2022. These included terms such as *“Delivery complications, Birth Doula Care, Perception of childbirth pain, Underwater birth, Pain experienced during childbirth, Postoperative effects of cesarean section, Lamaze breathing birth, Labor pain, Process of cesarean delivery, Epidural, Amniotic sac, Why not promote ELA, Pain associated with cesarean section, Cesarean delivery, The effects of ELA, The cost of ELA, Comprehensive parenting resources, Patient controlled analgesia, Antepartum pain.”* Only a small number of these high-frequency terms – such as “epidural, why not promote ELA, the effects of ELA, and the cost of ELA” – were directly relevant to ELA. This pattern demonstrates that the majority of public searches associated labor pain management with general childbirth practices or alternative delivery methods rather than specifically with ELA.

When narrowing the focus to the top 10 search terms (Fig. 3B), a similar trend was observed. The leading queries included *“Cesarean delivery, The effects of ELA, Underwater birth, Vaginal delivery, Why not promote ELA, The cost of ELA, Episiotomy, Comparison between vaginal delivery and cesarean delivery, Birth doula care, Pain experienced during childbirth.”* In contrast, only 3 of the top 10 terms – the effects of ELA, the cost of ELA, and why not promote ELA – directly addressed ELA itself. This imbalance highlights that while some internet users demonstrate explicit curiosity about ELA, public attention is still dominated by broader concerns about childbirth or general labor pain.

Taken together, these findings indicate that public awareness of ELA remains fragmented and limited. Queries such as “why not promote ELA” and “does ELA hurt during childbirth” suggest uncertainty and misconceptions, while the dominance of general childbirth-related searches points to a knowledge gap regarding the role and benefits of epidural analgesia. These findings underscore the need for targeted health education and professional communication strategies to provide accurate information, promote ELA, and address the issues most relevant to the public.

### 3.5. Impact of key events on public search behavior

To investigate the potential impact of the “Notice” and the maternal suicide incident on public concern about ELA, we first assessed the distribution of the BDI data. The Kolmogorov–Smirnov test confirmed that the BDI data for all analyzed periods were not normally distributed (all *P* < .001, see Table S1, Supplemental Digital Content, https://links.lww.com/MD/Q685 for full details of K–S statistics and *P*-values for each period). This justified the use of nonparametric tests for subsequent comparisons.

We then compared the BDI values for these 2 events before and after a 6-month period, excluding the 30 days immediately following the events to mitigate the influence of short-term extreme data fluctuations. The Mann–Whitney *U* test revealed that public search behavior changed significantly following both events (detailed results in Table S2, Supplemental Digital Content, https://links.lww.com/MD/Q685).

**Figure 4.**
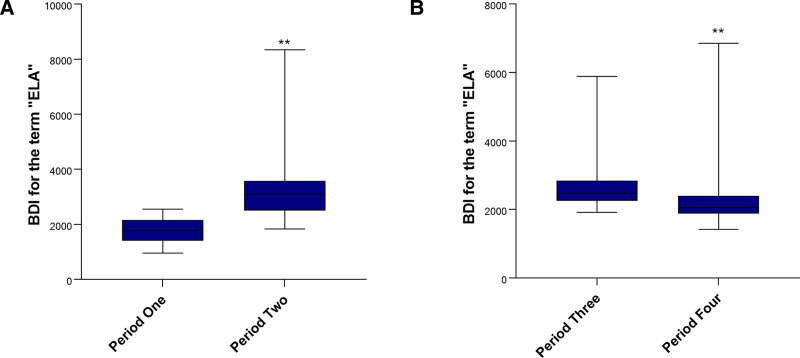
The influence of the Notice (A) and maternal suicide due to childbirth pain (B) on BDI with respect to ELA. The study was divided into 4 periods for comparison: period 1 (May 20, 2018–November 19, 2018), period 2 (December 21, 2018–June 19, 2019), period 3 (March 1, 2017–August 30, 2017), and period 4 (October 1, 2017–March 31, 2018). The Mann–Whitney *U* test was used to compare the data for (A, B) considering the non-normal distribution of the data. Statistical significance was denoted by * *P* < .05 and ** *P* < .01. BDI = Baidu index, ELA = epidural labor analgesia.

The BDI for ELA significantly increased after the release of the “Notice” compared to before its issuance (3096 [2505–3579] vs 1786 [141–2158]; *U* = 1.93 × 10⁹, *P* < .001), underscoring the potent role of top-down policy guidance in sustainably elevating public discourse and interest in ELA (Fig. 4A). On the other hand, compared to before the maternal suicide incident, the average BDI for ELA displayed a downward trend following the incident (2055 [1884–2397] vs 2471 [2254–2842]; *U* = 2.72 × 10⁹, *P* < .001; Fig. 4B). Based on the current results, the apparent surge in public attention following negative events may be superficial, overshadowed by their more profound adverse consequences. Although such incidents initially stimulate public discourse and information-seeking behavior, this heightened engagement is often transient and potentially counterproductive. More significantly, these events may fail to foster sustained interest in the specific medical procedure itself, while simultaneously creating strong mental associations with adverse outcomes. Ultimately, this dynamic may lead to a net decline in constructive public inquiry over time.

## 4. Discussion

The development of the internet has resulted in increased public dependence on Baidu as a primary source of health-related information. The prospect of applying data from the Baidu search engine to medical research topics is promising. This source of data may also serve to expand and supplement existing clinical and epidemiological data.^[18,36,37]^ Furthermore, the assessment of the search behaviors of internet users might guide policy planning and communication.^[18,36]^ Baidu boasts the largest market share of any search engine in China.^[38]^ BDI is officially launched in 2007, and BDI version 2.0 is released in 2013, which attracts the attention of the whole network and has been widely used in big data analysis in the fields of finance, tourism, health, etc. The present study aimed to identify and explore the interest of internet users in ELA using the BDI.

Our results demonstrated that from 2016 to 2018, the search volume for the term “ELA” exhibited a fluctuating upward trend over time, followed by a decline in the first half of 2018 and subsequent growth. However, after 2019, the search volume displayed a downward trend over time. There were 2-time points worthy of attention, including August 2017 and November 2018. The Notice release and the maternal suicide incident caused searching peaks. The first peak did not last very long, whereas the second peak lasted for more than 6 months. Our statistical comparisons, however, reveal a more nuanced story. Contrary to what might be expected from a transient peak, the Mann–Whitney *U* test confirmed a significant and sustained increase in search volume in the 6 months following the “Notice” compared to the period before it (*U* = 1.93 × 10⁹, *P* < .001; Table S2, Supplemental Digital Content, https://links.lww.com/MD/Q685). This robustly confirmed the role of national policy guidance in promoting public interest in ELA. Conversely, the maternal suicide incident was associated with a statistically significant decrease in search volume in the subsequent 6 months (*U* = 2.72 × 10⁹, *P* < .001; Table S2, Supplemental Digital Content, https://links.lww.com/MD/Q685), suggesting its impact was profoundly different from the policy-driven surge.

ELA technology in China has gradually improved and reached international standards. However, the current implementation of ELA in China is not optimistic, and its promotion is still facing great challenges. There are many reasons for the current limitations of ELA in China.^[15,39,40]^ First, many are influenced by the traditional Chinese concept that pain is part of the natural course of pregnancy. Second, the publicity for ELA is inadequate. Many women, including female journalists, do not know whether ELA is safe for women and infants. Third, ELA is given little attention in China. New technologies in obstetrics, such as ELA, have not been included in medical insurance catalogs. Fourth, the partners of anesthesiologists, including obstetricians and midwives, still have misunderstandings about ELA. Finally, there is still a shortage of anesthesiologists in China.

Nevertheless, the BDI searching trends revealed in our study might also reflect some promising characteristics of online public attentions. We found that the average value of the mobile search index for ELA was higher than that of personal computers, possibly due to the advancements in internet and mobile device technology in recent years. This may allow doctors and educators to better guide target patients ELA through behavioral and habitual intervention for ELA. The patients are increasingly using mobile devices to obtain relevant information about ELA. According to our spatial distribution analysis, the top regions, cities, and provinces with the highest search volumes for ELA were characterized by having a large population, public health awareness, good medical conditions, perfect internet infrastructure, and a high socioeconomic level. Most of the searches for ELA came from women (62.62%; Fig. 2A) and the group aged 20 to 39 years (81.46%; Fig. 2B). We hypothesized that women aged 20 to 39 represented the main group experiencing childbearing.

The relevant search terms identified from the BDI can be applied in healthcare to provide education and inform medical practice. The 10 relevant search terms with the highest frequencies suggested that most internet users had a poor understanding of ELA. Except for ELA, the other top 20 terms, many were only indirectly related to ELA, such as “delivery complications,” “birth doula care,” “underwater birth,” and “comprehensive parenting resources.” These terms suggest that internet users often associate labor pain management with general childbirth practices rather than specifically with ELA (Fig. 3A). Even within the top 10 search terms, which included “cesarean delivery,” “episiotomy,” and “pain experienced during childbirth,” the focus was largely on alternative delivery methods and general concerns about labor pain. In contrast, only a few terms, such as “the effects of ELA,” “the cost of ELA,” and “why not promote ELA,” directly addressed ELA itself (Fig. 3B). This pattern indicates that while some users are aware of ELA, the majority of searchers reflect uncertainty, misconceptions, or a lack of specific knowledge. Therefore, the search term data highlight a substantial gap in public understanding and emphasize the need for targeted health education by anesthesiologists, gynecologists, and health educators to provide accurate information, promote ELA, and address the issues that the public is most concerned about. This imbalance reinforces our conclusion that public understanding of ELA remains fragmented and highlights the critical role of targeted education in bridging this knowledge gap. Furthermore, although this study focused on mainland China, the methodology of using online search data to track public awareness of labor pain management has international relevance. Similar approaches could help other countries identify gaps in public knowledge and design educational or policy interventions tailored to their populations.

Our study also found that public concerns regarding ELA increased after the release of the Notice, demonstrating the significant guiding role of national policies in promoting ELA. The statistically significant increase in searches (*P* < .001) provides concrete evidence for this effect. The 1-child policy was first implemented in 1980 to control the population size in China.^[41]^ In recent years, the Chinese government has become aware of the problems associated with a low population growth rate. China’s low fertility rate is often regarded as a major factor affecting economic and social development.^[42]^ Therefore, the 2-child and 3-child policies have gradually emerged and become enforced.^[41,43]^ However, the increase in fertility can make childbirth progressively more difficult, as seen in developed countries.^[43]^ ELA can help to prevent women from choosing CD due to fear of pain, thus promoting maternal and infant safety. ELA also allows pregnant women to avoid many unpleasant aspects of childbearing. Therefore, we speculated that both the promotion and implementation of ELA were conducive to improve fertility desire. Policy factors can greatly increase the public attention to ELA. Therefore, the Chinese government should develop and strengthen policies to guide, promote, and implement ELA.

From a methodological standpoint, our reliance on nonparametric tests and median (IQR) reporting was necessitated by the nature of the data. The Kolmogorov–Smirnov tests confirmed that BDI values across all study periods were not normally distributed (all *P* < .001; Table S1, Supplemental Digital Content, https://links.lww.com/MD/Q685). This characteristic of real-world behavioral data underscores the importance of using robust statistical methods in infodemiology studies to draw valid conclusions about public attention patterns.

There were several worth noting limitations in this study. First, this study was carried out based on the internet, thus ignoring the search behaviors of people who live in areas with limited internet access. According to statistics from the China Internet Network Information Center (CNNIC), the internet was available to 70.4% of all Chinese citizens as of December of 2020. Second, other factors that were most likely associated with search behaviors, such as urbanization status, socioeconomic status, educational background, ethnicity, and online search motivation, were not considered. Further research is required to assess how these factors complicate the use of the BDI for estimating the status of search behaviors involving ELA. Third, this study did not evaluate the data generated by other search engines with low utilization, such as 360 and Shenma.

## 5. Conclusions

In this study, we used the BDI to monitor Chinese public interests in ELA. We found that Chinese internet users did not give much attention to ELA, and demonstrated a limited and fragmented understanding of the topic, as most related search terms reflected general childbirth practices and pain management rather than specific knowledge of ELA. As exemplified by the Notice, policy factors might greatly increase the public interests in ELA the topic, highlighting the role of government actions in shaping public awareness. The policies might effectively lead, promote, and implement ELA. Additionally, professional medical personnel and media should disseminate accurate knowledge and evidence-based guidance on ELA through online platforms and social media to support national policies, while enhancing health education initiatives to address prevalent public concerns such as cost, effectiveness, and safety of ELA. Ultimately, more efforts are needed to improve ELA use in China by integrating policy measures with public health education and professional advocacy. Beyond China, this study also demonstrates the value of infodemiology tools such as the BDI for identifying knowledge gaps in maternal health. By revealing public misconceptions and priorities, such analyses can guide both national and international strategies to improve labor pain management and maternal well-being.

## Author contributions

**Data curation:** Xinzhu Zhu, Xu Wang, Xuetong Zhang.

**Formal analysis:** Xinzhu Zhu.

**Methodology:** Xinzhu Zhu, Xu Wang, Xuetong Zhang.

**Software:** Xinzhu Zhu, Xu Wang, Xuetong Zhang, Peiwen Jia.

**Supervision:** Guannan He, Fengshou Chen.

**Validation:** Xinzhu Zhu, Xu Wang, Xuetong Zhang, Guannan He, Fengshou Chen.

**Visualization:** Guannan He, Fengshou Chen.

**Writing – original draft:** Guannan He, Fengshou Chen.

**Writing – review & editing:** Guannan He, Fengshou Chen.

## Supplementary Material


