# Supplementary material for: Public attention related to epidural labor analgesia in mainland China: Evidence from internet search data
Source: Medicine (Baltimore). 2025 Nov 14;104(46):e45978. doi: 10.1097/MD.0000000000045978 (PMC12622705; doi:10.1097/MD.0000000000045978)
Supplement: Supplementary file 1 [file medi-104-e45978-s001.docx]

**Supplementary Table 1.** Results of the Kolmogorov-Smirnov (K-S) Normality Test for Baidu Index (BDI) Data.

| **Period** | **Timeframe** | **KS Statistic** | **p-value** | **Normality conclusion** |
| --- | --- | --- | --- | --- |
| Full Period | 2016-01-01 to 2024-10-16 | 0.350 | <0.001 | Not normally distributed |
| Period One | 2018-05-20 to 2018-11-19 | 0.296 | <0.001 | Not normally distributed |
| Period Two | 2018-12-21 to 2019-06-19 | 0.298 | <0.001 | Not normally distributed |
| Period Three | 2017-03-01 to 2017-08-30 | 0.306 | <0.001 | Not normally distributed |
| Period Four | 2017-10-01 to 2018-03-31 | 0.299 | <0.001 | Not normally distributed |

**Supplementary Table 2.** Results of Mann-Whitney U Tests Comparing BDI Before and After Key Events.

| **Comparison** | **Groups (Timeframe)** | **U statistic** | **p-value** | **Conclusion** |
| --- | --- | --- | --- | --- |
| Notice (before vs. after) | Period One (2018-05-20 to 2018-11-19) vs. Period Two (2018-12-21 to 2019-06-19) | 1.9336 × 10⁹ | <0.001 | Significant increase in BDI after the Notice |
| Maternal suicide (before vs. after) | Period Three (2017-03-01 to 2017-08-30) vs. Period Four (2017-10-01 to 2018-03-31) | 2.7230 × 10⁹ | <0.001 | Significant decrease in BDI after the incident |

**Supplementary Figure 1.** The Baidu Search Volume Index in mainland China for the term "ELA” was obtained from January 1, 2016, to October 16, 2024. The symbol "#" represents the first peak node, while the symbol "@" represents the second peak node.
